# Survey of professionals on breast cancer, fertility preservation and pregnancy in Argentina

**DOI:** 10.3332/ecancer.2021.1183

**Published:** 2021-02-10

**Authors:** Alejandra García, Gabriela Candás, Agustina Bemi, Héctor Daniel Vuoto, Ernesto Korbenfeld, Juan Isetta, Lucas Cogorno, Agustina González Zimmermann, Marcia Sigal, Santiago Acevedo, Julia Berwart, Martín Naveira, María Delfina Ocampo, Juan Luis Uriburu

**Affiliations:** 1 Mastology Service, British Hospital of Buenos Aires, Perdriel 74, CABA, Buenos Aires C1280AEB, Argentina; 2 Oncology Service, British Hospital of Buenos Aires, Perdriel 74, CABA, Buenos Aires C1280AEB, Argentina; 3 Mastology Service, British Hospital of Buenos Aires, Perdriel 74, CABA, Buenos Aires C1280AEB, Argentina

**Keywords:** survey, medical knowledge, breast cancer, preservation of fertility, breast cancer associated with pregnancy

## Abstract

**Introduction:**

Medical knowledge regarding preservation of fertility and pregnancy in patients with breast cancer (BC) is of interest. We, therefore, decided to conduct a survey on this issue among professionals involved in the treatment of BC in Argentina.

**Materials and methods:**

A survey was conducted and sent by email to 3,412 contacts in the Argentine Mastology Society (Sociedad Argentina de Mastología, or SAM) database, with responses from 396 physicians. The survey design was based on the Lambertini 2017 survey. To the author’s knowledge, it is the first Argentine survey to address this issue.

**Results:**

The frequency with which the impact of cancer treatment on the fertility of young patients was addressed by the respondent and referred to a fertility specialist was ‘always’ and ‘almost always’ in 86.8% and 78.5% of cases, respectively.

**Conclusions:**

The level of knowledge is comparable to the data presented by other surveys. Membership in a Mastology Unit was associated with more current treatment. Continued work on the training of professionals is necessary to facilitate communication, information and guidance of patients of childbearing age who are going to have cancer treatment in order to advise them on fertility preservation, as well as the possibility of pregnancy after diagnosis of BC, and to be able to provide better care to those with BC associated with pregnancy.

## Introduction

In the United States from 2013 to 2017, the incidence of breast cancer (BC) in women aged 20–34 years represented 1.9% of diagnoses, and those aged 35–44 years represented 8.3%. The mortality rates were 0.9% in women aged 20–34 years and 4.6% for those between 35 and 44 years of age [1].

In a collaborative study published in 2016 that included 1,732 patients with BC in Argentina, the incidence by age was 7.4% for those between 30 and 39 years old and 8.5% for those between 40 and 44 years old [2]. According to data from the Breast Cancer Registry (Registro de Cáncer de Mama, or RCM) of the Argentine Mastology Society (SAM), 10.4% of infiltrating carcinomas were found in young women [3].

Globally, deaths from BC have been declining since the late 1980s in both young and older women [4]. According to the statistics published by the Surveillance, Epidemiology and End Results Program (SEER) [1], the age-adjusted rate of new BCs has increased 0.3% per year in the last 10 years, while mortality decreased 1.5% annually from 2008 to 2017. The 5-year survival rate has increased, reaching 91.27% in 2013.

Due to the increased survival of patients and the delay of motherhood for personal and social reasons, it is likely that young women diagnosed with BC have not fulfilled their plans for motherhood at the time of diagnosis, and are interested in their future fertility as well as the possibility of facing BC during pregnancy.

The first doctors to come into contact with young women with BC, whether they are mastologists, gynaecologists or oncologists, must know the needs and particular characteristics of this population. It is they who will have the opportunity to make a timely referral to a fertility specialist, as well as choosing the best therapeutic strategies for those with a diagnosis of BC during pregnancy to carry the foetus to term, without compromising the oncological safety of the mother or vitality and normal development of the foetus.

Today there are international guidelines with different algorithms for the management of young patients who wish to preserve their fertility [4–6], as well as the management of BC during pregnancy [7, 8], with the aim of providing up-to-date evidence to support medical practice.

According to a recent publication, using data from American Association of Clinical Oncology’s (ASCO) Quality in Oncology Practice Initiative [9], only 56% of women received fertility preservation counselling. According to the guidelines of the ASCO, the National Comprehensive Cancer Network and the European Society for Medical Oncology (ESMO) [5–7], consultations with pre-menopausal patients with BC should include fertility preservation counselling prior to beginning treatment, and no patient should be excluded.

Medical knowledge on these topics is of interest; therefore, surveys have been conducted on this topic in different countries in order to train and educate physicians, thereby improving the quality of advice and care for patients [10–13].

We, therefore, decided to conduct a survey of professionals involved in the treatment of BC in order to analyse the knowledge and current practice in the preservation of fertility and pregnancy in Argentina.

## Materials and methods

A survey (Appendix A) was conducted and sent via email to 3,412 contacts in the Argentine Mastology Society (SAM) database. It was sent three times: 31 July, 25 October25 and 17 December 2019. Five hundred and sixty-one professionals (16.4%) clicked on the link, and 396 physicians completed the survey (11.6% of the total emails sent, and 70.5% of those who clicked on the link).

Participation was anonymous and voluntary; consent was implicit when choosing to open the link in the email, which indicated its content (Appendix B).

The survey design was based on Lambertini *et al*. 2017 [13]. It was modified and adapted, and was divided into four sections. The first section included 11 questions on demographic data. A second section included 11 questions on BC and fertility preservation. The third section included 6 questions about pregnancy after BC, and finally, a fourth included 11 questions about BC during pregnancy. The survey was directed to different specialists involved in the care of patients with BC.

To the author’s knowledge, this is the first Argentine survey that addresses issues in the management of and current practices in BC, preservation of fertility and pregnancy, by the professionals who care for these patients.

### Statistical analysis

The category variables are described using the raw number and the percentage in each category. The Chi square test was performed with Mantel-Haenszel correction. The *p* value for statistical significance was <0.05. The OpenEpi program, version 3.01, was used to perform the calculations.

## Results

Three hundred and ninety-six responses were received.

### Demographic characteristics

62.4% of those surveyed were between 30 and 49 years of age. 87.7% were gynaecologists or mastologists, 4.5% were clinical oncologists and 91.4% said that they would be interested in receiving information on the topic (Table 1).

### Fertility preservation

**Figure 1.**
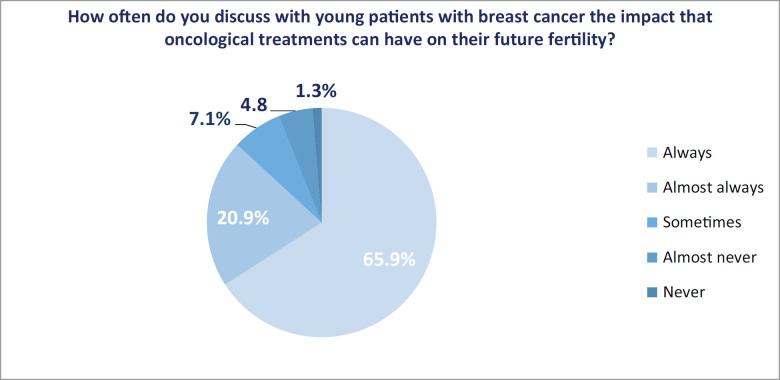
Discussion of the impact of oncological treatment on future fertility.

**Figure 2.**
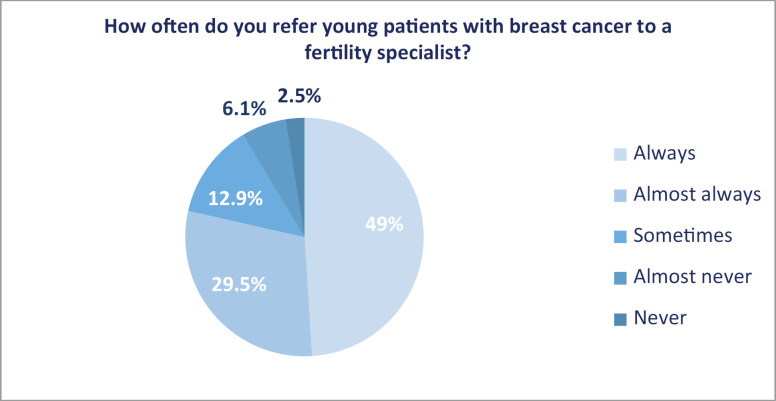
Frequency of referral to a fertility specialist.

The impact of cancer treatment on the fertility of young patients was addressed and referred accordingly ‘always’ and ‘almost always’ in 344 respondents (86.8%) and 311 (78.5%), respectively (Figures 1 and 2).

**Figure 3.**
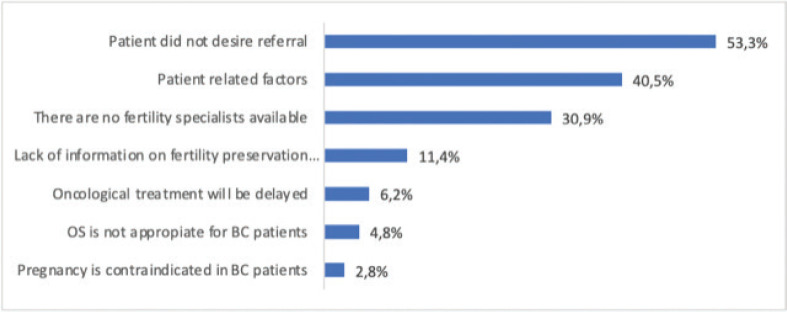
Reason for not referring patients to a fertility specialist. (n: 210). OS = Ovarian stimulation; pt = patient; BC = breast cancer.

In 210 cases, a response was provided for the most frequent reasons why patients were not referred to a fertility specialist: the patient did not desire referral 112 (53.3%); factors related to the patient: age, parity, marital status, cancer prognosis 85 (40.5%); there are no fertility specialists in their area 65 (30.9%); and lack of information on available techniques 24 (11.4%) (Figure 3).

We did not find statistically significant differences when evaluating the approach to the impact of cancer treatment on fertility (*p* = 0.6) and referral (*p* = 0.9), according to the speciality of the respondent: gynaecologist: always or almost always discuss 86.8% and provide referral 80.6%; mastologist: 90.1% and 79.6%; and oncologist: 94.4% and 77.8%.

**Figure 4.**
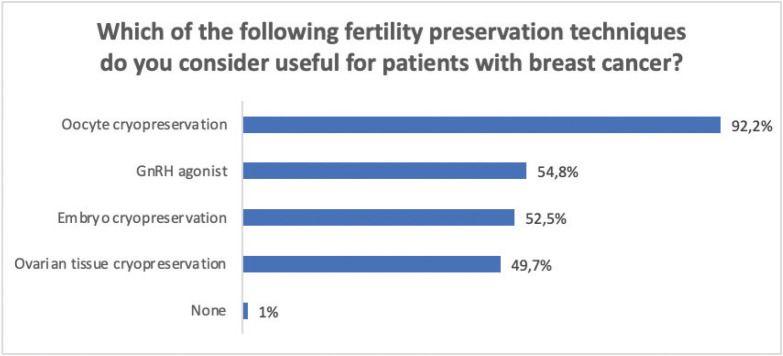
Knowledge regarding utility of different techniques of fertility preservation in patients with BC. BC = Breast cancer; CT = chemotherapy.

Regarding fertility preservation techniques, the majority, 365 (92.2%) considered oocyte cryopreservation useful (Figure 4).

The responses regarding the safety of ovarian stimulation (OS) and the impact of cancer treatments on fertility are detailed in Table 2.

### Pregnancy after BC

When evaluating the risk posed by pregnancy after BC, 66.2% of those surveyed affirmed that the risk does not increase, 45.2% considered that it does not do so within the first 2 years of diagnosis or with positive oestrogen receptor (ER) disease (58.3%) (Table 3).

51.3% considered it safe to temporarily interrupt hormone therapy (HT) after 18–30 months to attempt a pregnancy.

### BC associated with pregnancy

39.9% of those surveyed answered that BC during pregnancy, even when treated properly, has a worse prognosis. 33.6% stated that patients diagnosed at 28–33 weeks should be indicated a preterm delivery (PD) to begin cancer treatment (Table 4).

Regarding the identification of the sentinel node, 28.5% and 52% considered the use of patent blue and radiocolloids safe, respectively.

When evaluating the safety of the different adjuvant treatments, we see that 66.4% considered chemotherapy (CT) safe from the second trimester on; 85.6%, 74.7% and 62.1% did not consider the use of radiation treatment (RT), HT and anti-HER2 therapies to be safe.

### Evaluation according to membership in a Mastology Unit (MU)

13.8% (*n* = 15) of the professionals who work in a Mastology Unit (MU) do not have fertility specialists in their area, nor do 17.4% (*n* = 50) of those who do not work in a MU; this difference was not statistically significant (*p* = 0.7) (Table 5).

Eighteen questions were selected according to the clinical relevance of the knowledge. The responses were analysed, taking into consideration whether or not the respondent belonged to a MU and whether they were in accordance with current guidelines and recommendations [4–8].

We found a statistically significant difference in those professionals who belonged to these units in 16 of the 18 questions evaluated.

## Discussion

The future fertility and oncological safety of a patient with a pregnancy following BC diagnosis are important issues for many young women [14]. The knowledge of the physicians who treat these patients is essential to provide adequate information in the decision-making process and improve the quality of care.

### Fertility preservation

In our survey, we observed that 86.8% of treating physicians address the impact that cancer treatment will have on the fertility of young patients. Although it is an important percentage, it was lower than that published in the literature, which reports values between 91.6% and 98% [11–14].

Referral to a fertility specialist has evolved over time: in the surveys conducted by Quinn in 2009 [10] and Forman in 2010 [11], it was specified in only 47% and 39%. The inclusion of this topic in the guidelines surely contributed to a favourable change, as evidenced by Adams in 2013 [12], where referral reached 67%, reaching 97% according to Rosenberg in 2017 [14]. In our study, referrals were given in 78.5% of cases, and although this is an overall improvement, it remains sub-optimal.

The most frequent reason for lack of referral was that the patient did not want it (53.3%), and factors related to the patient: age, parity, marital status, cancer prognosis (40.5%), similar to that published by Lambertini *et al* [13], who found that these factors are the main barrier to accessing consultation with a fertility specialist (53.8%). However, according to Forman *et al* [11], these factors should not deprive a patient of the discussion regarding fertility preservation.

When evaluating patient surveys by Ruddy [15] and Partridge [16], future fertility is a matter of great concern, which may even impact their therapeutic decisions [17, 18]. Therefore, it is striking that one of the most frequent causes of lack of referral is that the patient does not want it. It is worth questioning how we inform patients on this topic, and how our beliefs, opinions or lack of knowledge impact that decision, as, according to that published by Peate *et al* [19], 75% of the patients base their decision on the opinion of their doctor.

The absence of fertility specialists and the lack of information on available techniques were other causes of the lack of referral, data that coincide with that published by Lambertini (30.9% versus 28.2% and 11.4% versus 17.9%) [13]. It must be mentioned that in Argentina, public and private institutions have their own fertility services or access these services following the concept of MUs without borders.

It is encouraging that 91.4% of the respondents wish to receive information on the subject and 72% would attend a course, similar to that published by Forman [11].

In the study conducted by Masciello *et al* [20], 78% of mastologists prefer to defer the discussion about fertility preservation for the Oncologists to address. On the other hand, we did not find statistically significant differences between the different professionals involved.

Nearly all of the respondents (92.2%) considered oocyte cryopreservation, one of the currently most recommended techniques, to be useful [5]. However, a lack of knowledge of different techniques, between 7.8% and 50.3%, is observed.

More than half of those surveyed did not consider OS safe or were unaware of it, especially in those with ER positive BC, similar to the 43.2% published by Lambertini *et al* [13]. Regarding OS protocols in BC patients, 51.3% stated that they should include letrozole or tamoxifen, similar to that described by Sanada *et al* [21], who used aromatase inhibitors in up to 68.3% of patients with BC. The results of the prospective STIM trial (NTR4108) confirming the safety of these protocols are awaited [22].

62.9% considered that the use of gonadotropin-releasing hormone (GnRH) analogues during CT could be offered to all pre-menopausal patients with BC who wish to preserve their fertility, regardless of ER status. Randomised studies [23, 24] and meta-analyses [25] evaluated the efficacy of this procedure even in ER positive patients. ASCO 2018 [5] and the Consensus of Breast Cancer in young women (BCY4) [4] consider that this method should be offered to patients who are interested in fertility preservation, and who cannot or do not wish to use other methods. Lambertini *et al* [25] suggests that they would not be exclusive and could be offered to patients who have already used other preservation techniques. Ovarian suppression was the best known strategy 82.4%, in the Lambertini survey [13]; however, in our study, only 54.8% considered it useful. This could be related to the lack of coverage of other techniques in some countries.

It is not necessary for all professionals to have a thorough understanding of every fertility preservation procedure. However, the patient of childbearing age who is going to receive cancer treatment should be informed about safety and the possibility of consultation with a fertility specialist to advise her on the different options available. It is important to acknowledge to the patient that it is desirable to preserve her fertility and to make a rapid referral.

### Pregnancy after BC

In evaluating the risk posed by pregnancy after BC, 66.2% of those surveyed stated that it does not increase, 45.2% considered that it does not do so within the first 2 years of diagnosis, or in ER positive disease (58.3%). In his survey, Lambertini [13], reported similar data: 69.6%, 50.5% and 63%, respectively. Current evidence suggests that pregnancy after BC does not increase the risk of relapse [4]. Some 62.4% of the professionals considered assisted reproductive techniques safe in a patient with a history of BC, similar to the 57.5% reported in the literature [13].

The best time to become pregnant remains controversial. Experts suggest waiting 2 years after diagnosis to start trying become pregnant [26].

In our survey, 51.3% responded that it is safe to temporarily interrupt hormone treatment after 18–30 months to allow a pregnancy, as did the 50.5% reported by Lambertini [13]. This behaviour is supported by the BCY4 Consensus [4] and Saint Gallen 2019 [27]; however, it will be the results of the POSITIVE study (IBCSG 48-14 NCT02308085) that provides the answer on regarding its safety [28].

### BC during pregnancy

Despite the fact that BC during pregnancy does not represent a worse prognosis for women [8, 29], 39.9% of those surveyed answered yes, even when it is treated appropriately, similar to the 31.5% reported in the literature [13]. It is worth mentioning that distinct publications found that a postpartum BC diagnosis may be associated with a worse prognosis than a diagnosis during pregnancy [30].

33.6% stated that in patients diagnosed at 28–33 weeks, a preterm birth is indicated at the start of oncological treatment; however, the greatest risk to consider is prematurity. The best option, when possible, is to carry out treatments during pregnancy and carry to term [7, 8].

In accordance with current evidence, sentinel node biopsy (SNB) is a procedure that can be carried out in a safe manner during pregnancy [7, 8]. For studies using radiocolloids, some 52% of the respondents considered the use of radiocolloids safe during pregnancy; on the other hand, 55.8% contraindicated patent blue due to the potential for anaphylactic reactions [7, 8, 31].

In evaluating the safety of different adjuvant treatments, it is seen that 66.4% considered CT to be safe to start in the second trimester, which is correct, and that its use during the first trimester is associated with foetal malformations and miscarriage [7, 8].

85.6%, 74.7% and 62.1%, respectively, responded that radiation therapy, HT and anti-Her2 treatment should be avoided during pregnancy. Lambertini [13] published similar numbers—76.6%, 76.2% and 61.9%.

### Knowledge according to belonging to a MU

MUs are created to optimise the quality of patient care through better coordination and communication between different disciplines, and for advances in knowledge to be applied in a uniform manner, benefiting every patient and professional in the MU.

In 2015 [32], the SAM developed an accreditation programme based on international guidelines [33, 34]. In the completed survey, it is evident that belonging to a MU leads to enrichment and continuing education, such that the responses of the professionals who engaged in these were in accordance in a statistically significant manner with available evidence [4, 5, 7, 8, 27, 35–38].

In order to carry out this work, the SAM database of professionals was used. The response rate was low, though similar to other surveys [10, 12]; thus conclusions could not be extrapolated to what is happening throughout Argentina. Other limitations of the study were selection bias, which is possible given that only those professionals who are interested in the topic decided to answer.

Its strength is that it is the first study to permit evaluation of current knowledge and practices in relation to the topics in this field and the great interest that they generate.

## Conclusion

Based on these results, we can conclude that the level of knowledge is comparable to the data presented in other surveys. Belonging to a MU was associated with being more up-to-date on this topic. It is necessary to continue working to train professionals in facilitating communication, information and advising patients of childbearing age who are going to receive oncological treatment so they can be informed about fertility preservation as well as the possibility of pregnancy following a BC diagnosis, and providing better care to those with BC during pregnancy.

## Conflicts of interest

The authors declare no conflict of interest related to the present work.

## Funding

Nothing of a financial nature was received for the execution of the present work.

## Figures and Tables

**Table 1.** Demographic characteristics of individuals polled.

	*n*	%
**Total population**	**396**	**100%**
**Age**		
20–29 years	18	4.5
30–39 years	121	30.6
40–49 years	126	31.8
50–59 years	67	16.9
60 or older	64	16.2
**Gender**		
Women	237	59.8
Men	159	40.2
**Speciality**		
Mastology	172	43.5
Gynaecology	175	44.2
Oncology	18	4.5
Obstetrics	17	4.3
General surgery	8	2
Other	6	1.5
**Usual place of practice**		
Private practice	77	19.4
Hospital/sanatorium	121	30.6
Both	195	49.2
Other	3	0.8
**Years in practice**		
1–5 years	71	17.9
6–10 years	62	15.6
11–15 years	67	16.9
16–20 years	56	14.1
20 years or more	140	35.5
**Work in a MU accredited by SAM**		
Yes	109	27.5
No	287	72.5
**Patients <40 years seen annually**		
Under 10	218	55.1
10–50	147	37.1
More than 50	31	7.8
**Patients with BC during pregnancy seen in the last 10 years**		
None	81	20.5
1–5	219	55.3
6–10	69	17.4
More than 10	27	6.8
**Interested in receiving information about the topic addressed**		
Yes	362	91.4
No	11	2.8
Maybe	23	5.8
**Interested in attending a course about the topic addressed**		
Yes	285	72
No	25	6.3
Maybe	86	21.7

MU: Mastology unit, ASM: Argentine Society of Mastology, PABC: pregnancy-associated BC

**Table 2.** Fertility preservation.

	Yes	No	NR
OS is considered safe in all patients with BC	180 (45.5%)	101 (25.5%)	115 (29%)
OS is considered safe in patients with ER positive BC	168 (42.4%)	98 (24.8%)	130 (32.8%)
OS is considered safe in patients who have received neoadjuvant CT	198 (50%)	69 (17.4%)	129 (32.6%)
OS in patients with a BC diagnosis should include letrozole or tamoxifen	203 (51.3%)	37 (9.3%)	156 (39.4%)
The use of analogues during CT can be offered to all pre-menopausal patients with BC who wish to preserve their fertility independent of ER status	249 (62.9%)	52 (13.1%)	95 (24%)
The impact of CT on future fertility depends on age, drugs and dosages used	350 (88.4%)	13 (3.3%)	33 (8.3%)
The risk of amenorrhoea following CT is greater in women < 30 years compared to women > 40 years	88 (22.2%)	245 (61.9%)	63 (15.9%)

OS: Ovarian stimulation, pts: patients, BC: breast cancer, ER: oestrogen receptors, CT: chemotherapy, NR: no response

**Table 3.** Pregnancy after BC.

	Yes	No	NR
Pregnancy after BC increases RR	77 (19.4%)	262 (66.2%)	57 (14.4%)
Pregnancy after BC increases RR only in the first 2 years following dx	127 (32.1%)	179 (45.2%)	90 (22.7%)
Pregnancy after BC increases RR only in ER positive cancers	64 (16.2%)	231 (58.3%)	101 (25.5%)
It is considered safe to temporarily interrupt HT after 18–30 months to allow a pregnancy	203 (51.3%)	79 (19.9%)	114 (28.8%)
Lactation after BC is considered safe	313 (79%)	22 (5.6%)	61 (15.4%)
ARTs are considered safe in patients who have had BC	247 (62.4%)	44 (11.1%)	105 (26.5%)

BC: breast cancer, RR: risk of recurrence, dx: diagnosis, ER: oestrogen receptors, HT: hormone therapy, ART: assisted reproduction techniques, NR: no response

**Table 4.** BC associated with pregnancy.

	Yes	No	NR
PABC, even when adequately treated, has a worse prognosis	158 (39.9%)	205 (51.8%)	33 (8.3%)
In patients diagnosed between 28 and 33 weeks, a PD is indicated	133 (33.6%)	208 (52.5%)	55 (13.9%)
Mx may be used with abdominal protection	368 (92.9%)	18 (4.6%)	10 (2.5%)
Conservative surgery may be considered, depending on the case	331 (83.6%)	45 (11.4%)	20 (5%)
SNB with patent blue may be used, depending on the case	113 (28.5%)	221 (55.8%)	62 (15.7%)
SNB with radiocolloids may be performed, depending on the case	206 (52%)	116 (29.3%)	74 (18.7%)
RT during pregnancy is safe	10 (2.5%)	339 (85.6)	47 (11.9%)
CT may be used safely in any trimester	7 (1.8%)	359 (90.6%)	30 (7.6%)
CT may be used safely from the second trimester on	263 (66.4%)	72 (18.2%)	61 (15.4%)
HT may be used safely	28 (7.1%)	296 (74.7%)	72 (18.2%)
Anti-Her2 treatment may be used safely	28 (7.1%)	246 (62.1%)	122 (30.8%)

PABC: Pregnancy-associated BC, dx: diagnosis, PD: preterm delivery, Mx: mammogram, SNB: sentinel node biopsy, RT: radiation therapy, CT: chemotherapy, NR: no response

**Table 5.** Knowledge according to belonging to a MU.

	MU Yes (*n*: 109)	MU No (*n*: 287)	
	AE agreement	AE agreement	*p*
Fertility preservation			
**Discussion of the impact of treatments on fertility** ASCO and BCY4 recommend addressing the subject of the impact that oncological have on the whole patient of childbearing age [4, 5]	103 (94.5%)	241 (84%)	**0.002**
**Referral to a fertility specialist** BCY4 recommends that all women interested in preserving their fertility should be referred to a specialist [4]	90 (82.6%)	221 (77%)	0.1
OS is considered safe in all patients with BC [35]	61 (56%)	119 (41.5%)	**0.004**
OS is considered safe in all patients with ER+ BC [35]	61 (56%)	107 (37.3%)	**0.0003**
OS is considered safe in patients who have received neoadjuvant CT [35]	69 (63.3%)	129 (44.9%)	**0.0005**
OS in patients diagnosed with BC should include letrozole and tamoxifen [35]	70 (64.2%)	133 (46.3%)	**0.0007**
Use of analogues during CT may be offered to all pre-menopausal patients with BC who wish to preserve their fertility independent of their ER status [25]	76 (69.7%)	173 (60.3%)	**0.04**
**Pregnancy after BC**			
Pregnancy after BC increases RR *Pregnancy after BC does not increase RR* [36]	85 (78%)	177 (61.7%)	**0.001**
Pregnancy after BC increases RR only in the first 2 years following dx *The interval between BC dx and pregnancy does not impact patient prognosis* [36]	54 (49.5%)	125 (43.5%)	0.14
Pregnancy after BC increases RR only in ER positive cancers *Pregnancy after BC does not increase RR, even in ER+ cancers* [36]	80 (73.4%)	151 (52.6%)	**0.00009**
It is considered safe to temporarily interrupt HT after 18–30 months to allow a pregnancy *The BCY4 and Saint Gallen guides recommend a minimum 18 months of HT before considering pregnancy, although prospective date is lacking* [4, 27]	66 (60.5%)	137 (47.7%)	**0.01**
**PABC**			
PABC, even when adequately treated, has a worse prognosis *Adequately treated PABC has the same prognosis as BC without pregnancy* [37]	71 (65.1%)	134 (46.7%)	**0.0005**
In patients diagnosed between 28 and 33 weeks, a PD is indicated *The ESMO guide* [7] *and different authors* [8, 38] *suggest that it is always preferable to carry a pregnancy to term*	69 (63.3%)	139 (48.4%)	**0.004**
SNB with patent blue may be used, depending on the case *The ESMO guide* [7]* and Loibl* [8] *note the contraindication for the use of patent blue during pregnancy*	79 (72.5%)	142 (49.5%)	**0.00001**
SNB with radiocolloids may be performed, depending on the case [7, 8]	81 (74.3%)	125 (43.5%)	**<0.0000001**
CT can be used safely from the second trimester on [7, 8]	97 (89%)	166 (57.8%)	**<0.0000001**
HT may be used safely *Tamoxifen is teratogenic and is found to be contraindicated during pregnancy* [7, 8]	96 (88.1%)	200 (69.7%)	**0.00008**
Anti-Her2 treatment may be used safely *Trastuzumab passes through the placenta from the second trimester on and is found to be associated with oligoamnios, and is therefore contraindicated* [7, 8]	81 (74.3%)	165 (57.5%)	**0.001**

MU: Mastology unit, AE: available evidence, tmts: treatments, OS: ovarian stimulation, PABC: pregnancy-associated BC, dx: diagnosis, PD: preterm delivery, Mx: mammogram, SNB: sentinel node biopsy, HT: hormone therapy, RT: radiation therapy, CT: chemotherapy

## Appendix A. Survey of current practices in BC, fertility preservation and pregnancy

**A) Demographic data**

1. Age: __________ years.

2. Gender: ____Female ____Male

3. What is your speciality?

____ Mastology ____ Oncology

____ Gynaecology ____ Imaging

____ General surgery ____ Radiotherapy

____ Obstetrics ____ Anatomical pathology

4. Are you a member of the ASM?

____Yes ____ Subscriber ____Member ____ Honorary ____ Lifetime Member

____ No

5. How would you define your regular practice?

____ Hospital/sanatorium

____ Private practice

____Both

____ Other (please specify): _____________

6. How long have you been in practice? ____________ years.

7. Do you work in a MU certified/accredited by AMS?

____ Yes

____ No

8. How many patients under 40 with BC do you treat annually?

____ fewer than 10

____ 10–50

____more than 50

9. How many patients with PABC have you seen in the last 10 years of practice?

____ 0

____ 1–5

____ 6–10

____ more than 10

10. Are you interested in receiving information on the topics in this survey?

____ Yes

____ No

____ Maybe

11. Are you interested in attending a course on the topics in this survey?

____ Yes

____ No

____ Maybe

**B) BC and fertility preservation**

1. How often do you discuss with the impact oncological treatment can have on future fertility with young patients with BC?

____ Always ____ Almost always ____ Sometimes ____ Almost never____ Never

2. How often do you refer young patients with BC to a fertility specialist?

____ Always ____ Almost always ____ Sometimes ____ Almost never____ Never

3. In cases wherein patients are not referred to a fertility specialist. Check ALL of the reasons you consider to be correct.

____ Lack of Information on available methods.

_____ No availability of fertility specialists in your work area.

____ You would not consider ovulation stimulation to be appropriate in these patients.

____ Consider that these procedures would delay starting cancer treatments.

____ You do not believe that it is advisable for patients with BC to attempt future pregnancies.

____ Patient-related factors: age, parity, marital status, cancer prognosis.

____ The patient does not want it.

____ Other reasons: Specify __________________________________________

4. Which of the following fertility preservation techniques are considered useful in patients with BC? Check ALL that you consider:

____ Embryo cryopreservation.

____ Oocyte cryopreservation

____ Ovarian tissue cryopreservation.

Use of GnRH analogues during CT.

____ None.

5. Check ALL statements with which you agree:

**Table:**

	Yes	No	No response
OS is considered safe in all patients with BC			
OS is considered safe in patients with ER positive BC			
OS is considered safe in patients who have received neoadjuvant CT			
OS in patients with a BC diagnosis should include letrozole or tamoxifen			
The use of analogues during CT can be offered to all pre-menopausal patients with BC who wish to preserve their fertility independent of ER status			
The impact of CT on future fertility depends on age, drugs and dosages used			
The risk of amenorrhoea following CT is greater in women < 30 years compared to women > 40 years			

OS; Ovarian stimulation, pts; patients, BC; breast cancer, ER; oestrogen receptors, CT; chemotherapy, NR; no response

**C) Pregnancy after BC**

**Table:**

	Yes	No	NR
Pregnancy after BC increases RR			
Pregnancy after BC increases RR only in the first 2 years following dx			
Pregnancy after BC increases RR only in ER+ cases			
It is considered safe to temporarily interrupt HT after 18–30 months to allow a pregnancy			
Lactation after BC is considered safe			
ARTs are considered safe in patients who have had BC			

BC: Breast cancer, RR: risk of recurrence, dx: diagnosis, ER: oestrogen receptors, HT: hormone therapy, ART: assisted reproduction techniques, NR: no response

**D) PABC**

**Table:**

	Yes	No	NR
PABC, even when adequately treated, has a worse prognosis			
In patients diagnosed between 28 and 33 weeks, a PD is indicated			
Mx may be used with abdominal protection			
Conservative surgery may be considered, depending on the case			
The SNB with patent blue may be performed, depending on the case			
SNB with radiocolloids may be performed, depending on the case			
RT during pregnancy is safe			
CT may be used safely in each trimester			
CT may be used safely from the second trimester on			
HT may be used safely			
Anti-Her2 treatment may be used safely			

PABC: Pregnancy-associated BC, dx: diagnosis, PD: preterm delivery, Mx: mammogram, SNB: sentinel node biopsy, RT: radiation therapy, CT: chemotherapy, NR: no response

Thank you very much for your responses and time.

## Appendix B. E-mail format used to request participation in the survey

Dear colleague:

We would like to request your assistance by responding to this anonymous survey about current practices in **breast cancer, fertility preservation and pregnancy**, the results of which will be used to conduct a scientific study. It will not take longer than 10 minutes to complete.

We appreciate your time and collaboration.

Sincerely,

Dr Gabriela Candás

Dr Alejandra García

Link to the survey

**If you have already completed the survey, please disregard this message. Thank you very much.**
